# Self-Triggered Formation Control of Nonholonomic Robots

**DOI:** 10.3390/s19122689

**Published:** 2019-06-14

**Authors:** Carlos Santos, Felipe Espinosa, Miguel Martinez-Rey, David Gualda, Cristina Losada

**Affiliations:** Electronics Department, University of Alcalá, Engineering School, Campus Universitario, 28871 Alcalá de Henares, Spain; felipe.espinosa@uah.es (F.E.); miguel.martinez@depeca.uah.es (M.M.-R.); david.gualda@uah.es (D.G.); cristina.losada@uah.es (C.L.)

**Keywords:** self-triggered Lyapunov control, real-time scheduling, practical stability, remote guidance, formation control, nonlinear trajectory tracking

## Abstract

In this paper, we report the design of an aperiodic remote formation controller applied to nonholonomic robots tracking nonlinear, trajectories using an external positioning sensor network. Our main objective is to reduce wireless communication with external sensors and robots while guaranteeing formation stability. Unlike most previous work in the field of aperiodic control, we design a self-triggered controller that only updates the control signal according to the variation of a Lyapunov function, without taking the measurement error into account. The controller is responsible for scheduling measurement requests to the sensor network and for computing and sending control signals to the robots. We design two triggering mechanisms: centralized, taking into account the formation state and decentralized, considering the individual state of each unit. We present a statistical analysis of simulation results, showing that our control solution significantly reduces the need for communication in comparison with periodic implementations, while preserving the desired tracking performance. To validate the proposal, we also perform experimental tests with robots remotely controlled by a mini PC through an IEEE 802.11g wireless network, in which robots pose is detected by a set of camera sensors connected to the same wireless network.

## 1. Introduction

Nowadays, multi-agent system is one of the most studied control topics; within this context, formation control has special relevance [1,2,3]. Advances in communication wireless technology and embedded devices, among other fields, have enabled the development of underwater robots [4], unmanned aerial vehicles [5] or terrestrial robots [2,3,6] working in formation. These groups of vehicles can properly deal with different challenges, such as navigation, mapping and monitoring works [2]. According to the way in which control actions are remotely applied to the units of the formation, centralized or decentralized control strategies can be defined. The centralized one is simpler in the design stage and is usually more robust against disturbances than decentralized alternatives because all units know the information of the rest of the units of the formation. However, its main disadvantage is that requires a large communication network to send all the information which makes its scalability limited [2,3]. On the contrary, decentralized control offers a solution to this problem [7,8] due to the control signal of each unit being computed according to its own state and the information of its neighbours, while at the same time guaranteeing the stability of the formation.

According to the mission carried out by the formation, three types of strategies can be differentiated [1]. When the position of each unit is measured with respect to a global coordinate system, this strategy is referred to as position-based control. If the unit measures the displacement of its environment to establish the formation, it is called displacement-based control [9]. Finally, when the units compute the formation, control actions according to the distance with the other units are categorized as distance-based control [3].

One of the main drawbacks in remote formation control is the bandwidth limitation of the communication network [10]. Other common shortcomings are time-varying delays [11,12], packet dropouts [13,14], packet disorder [13,15], and network bandwidth constraints [16,17]. These problems are especially important in the case under study, where the remote controller has to send and receive information about multiple sensor and actuator wireless nodes. To deal with these drawbacks, we propose the use of aperiodic control strategies due to the fact that they offer flexibility to adapt the transmission of sensory and control information through the wireless channel. Aperiodic control techniques employ information about the system state to decide when the control action must be updated. The main aperiodic control techniques are event-triggered (ETC) [18,19,20,21,22,23,24,25] and self-triggered control (STC) [26,27,28,29,30,31,32]. In ETC, the triggering mechanism is based on the constant measurement of the plant state. In contrast, STC predicts when the system will fulfil the triggering condition. In order to carry out the forecast of the future states of the plant, STC employs the latest measurement of the plant as well as its model. An extensive description of these controllers is presented in [33] and a comparative study is described in [34].

Several studies have reported aperiodic control of nonholonomic robots tracking nonlinear trajectories. In [2], a consensus control solution to establish a certain formation with a set of LEGO robots is presented. They use the ETC approach to guarantee asymptotic stability of the formation and to reduce information interchange through a Zigbee network. However, the main disadvantage is that each robot position captured by an external camera is continuously evaluated to check the triggering condition. In [18], an event-triggered controller is implemented to track a nonlinear trajectory with a Khepera III robot. A Lyapunov function supports the triggering mechanism working on the Cartesian reference system. As in the previous case, the main disadvantage is the periodic sending of sensory information. In [8], an event-based formation control scheme is designed for wirelessly linked mobile robots. The implemented formation control follows a distributed consensus strategy using neural networks; however, only simulation results are reported. Ref. [22] presents an event-triggered controller applied to a mobile robot wirelessly connected with a remote centre that computes the triggering condition based on the difference between the measured position and the reference one. When this error reaches a predefine threshold, a new event is generated. In [23], a framework is devised to control a vehicle platoon using event-based communication and nonlinear controllers. A decentralized triggering strategy is designed considering a platoon of heterogeneous vehicles with nonlinear dynamics. However, only simulation results are reported. We have also designed and implemented aperiodic remote controllers [3,19]. In [19], we combine event-based estimation with an event-triggered controller for the remote control of only one P3DX robot. In [3], we present a consensus strategy for the remote platooning guidance of autonomous vehicles; however, to simplify the problem, we assume longitudinal and lateral decoupling and we only present simulation results.

The main contributions of the present paper are:

1. Design and implementation of a novel self-triggered Lyapunov-based control for nonlinear systems, using a dual stability approach in order to guarantee practical stability. When the Lyapunov function is greater than a given threshold, asymptotic stability is guaranteed. After that, the system is bounded on the Lyapunov threshold level.
2. Evaluation of centralized and decentralized triggering mechanisms for formation control of nonholonomic robots tracking nonlinear trajectories, comparing both with a periodic implementation. The experimental set-up includes three mobiles robot remotely controlled in a scenario with four wireless camera sensors.
3. The design and implementation of a delay compensation strategy that leverages one of the main strengths of STC, namely that the next sampling instant is known in advance.

The paper is structured as follows: the problem statement is described in Section 2; the self-triggered Lyapunov control solution is presented in Section 3; Section 4 details the simulation results; in Section 5, the more relevant implementation aspects are described; the experimental outcomes are shown in Section 6; finally, Section 7 highlights the main paper contributions.

### Notation

We use the following notation: ||v|| is the Euclidean norm of the vector $v\in R^{n}$ and ∧ is the truth-functional operator of logical conjunction. A function is of class $C^{0}\left(D_{x}\right)$ if it is continuous over $D_{x}$, and it is $C^{l}\left(D_{x}\right)$, l>0 if its derivatives are of class $C^{l-1}\left(D_{x}\right)$. A continuous function ρ:[0,a)→+∞,a>0 is of class K if it is strictly increasing and ρ(0)=0. We represent a Lyapunov level set by $\Omega_{V_{k}}=\left\{\xi \left(t\right)\in R^{n_{x}}|V\left(\xi \left(t\right)\right)\leq V_{k}\right\}\subset D_{x}$.

## 2. Problem Statement

A common problem in remote vehicle guidance is the design of control laws to follow a time-parameterized reference. This problem is especially challenging when considering nonlinear trajectories and nonholonomic vehicles. Such is the case of differential-drive robots, which only possess two actuation variables (linear velocity and angular velocity) for locomotion control, whereas the pose of the mobile unit is characterized by three degrees of freedom. In [19], the ETC theory for one robot unit is already presented; here, an extension to an STC formation control for a group of robot units is proposed. We report the design and implementation of an aperiodic formation controller to carry out this task through a wireless network.

### 2.1. Formation Control Problem

**Figure 1:**
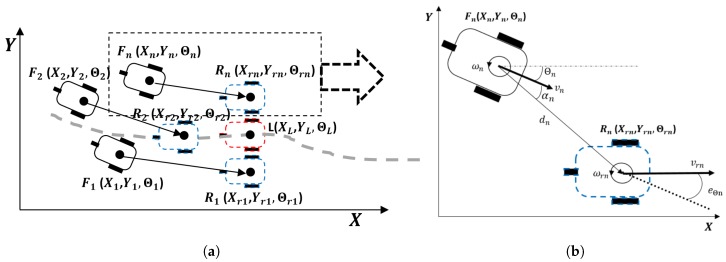
(**a**) main variables describing the trajectory tracking in formation strategy: in blue, the reference poses of each robot $R_{n}$ $\left(X_{rn},Y_{rn},\Theta_{rn}\right)$, which are generated according the position of a virtual leader robot *L* $\left(X_{L},Y_{L},\Theta_{L}\right)$ in red and in black the current position of each robot, described by $F_{n}$ $\left(X_{n},Y_{n},\Theta_{n}\right)$; (**b**) main variables describing trajectory tracking of one robot in the formation, where the robot reference is computed according to the position of the virtual leader: $d_{n}$ is the distance error calculated from the robot point $\left(X_{n},Y_{n}\right)$ to the reference point $\left(X_{rn},Y_{rn}\right)$, $\alpha_{n}$ is the orientation error with respect to the target point, $e_{\Theta_{n}}$ is the orientation error between the desired orientation to follow the trajectory $\left(\Theta_{rn}\right)$ and the orientation of the robot ($\Theta_{n}$).

The left picture of Figure 1 displays the principal elements of the formation, where the reference pose for each robot $R_{n}$ $\left(X_{rn},Y_{rn},\Theta_{rn}\right)$ is generated according to the position of a virtual leader robot *L* $\left(X_{L},Y_{L},\Theta_{L}\right)$ and the current position of each robot is described by $F_{n}$ $\left(X_{n},Y_{n},\Theta_{n}\right)$. The virtual leader tracks the nonlinear trajectory without error and is used to determine the position of each robot in the formation. To do this, we implement a road-following formation strategy to compute the position of each robot in each time and its velocity references.

Road-following formation allows two kinds of coordination: the spatial coordination and temporal coordination [35]. In the case of spatial coordination, the trajectories of each unit within the formation are coordinated, thus the shape of formation adapts to the conditions of the trajectory; therefore, the distances between the different agents of the formation can vary at certain times. On the contrary, in a temporally-coordinated formation, the distance between the different agents is permanently kept constant. Considering that our challenge is the nonlinear, trajectory tracking of a robot formation, we apply the spatial coordination despite the fact that this does not guarantee a constant relative position between the mobile units every time. For this reason, the trajectory generator is responsible for limiting the curvature of the trajectories andn establishing an equal linear velocity for all the units in the formation in order to avoid possible collision situations of the different units. To implement this trajectory generator, whose mission is to compute the position and velocity references of each real robot in the formation, we decided to use a virtual leader as a reference.

Figure 1a details the tracking problem for each robot in the formation. The current pose of each robot is characterized by $\left(X_{n},Y_{n},\Theta_{n}\right)$. The robot kinematics allows for establishing the relationship between pose time derivative and the related linear *v* and angular ω velocities. For the nonholonomic robot model, we have:

(1) $$\begin{array}{cc} {\dot{X}}_{n}\left(t\right) & =v_{n}\left(t\right)\;\cos\left(\Theta_{n}\left(t\right)\right),\\ {\dot{Y}}_{n}\left(t\right) & =v_{n}\left(t\right)\;\sin\left(\Theta_{n}\left(t\right)\right),\\ {\dot{\Theta }}_{n}\left(t\right) & =\omega_{n}\left(t\right). \end{array}$$

We also define these error variables for each agent (Figure 1b): angle error $\alpha_{n}$ is defined as the different between the current orientation and the orientation needed to reach the target point, orientation error $e_{\Theta_{n}}$ is the different between the desired ($\Theta_{rn}$) and actual ($\Theta_{n}$) orientations; finally, distance error $d_{n}$ is calculated as the distance between the current and reference positions:

(2) $$\begin{array}{cc} d_{n}\left(t\right) & =\sqrt{{\left(X_{rn}\left(t\right)-X_{n}\left(t\right)\right)}^{2}+{\left(Y_{rn}\left(t\right)-Y_{n}\left(t\right)\right)}^{2}}, \end{array}$$

(3) $$\begin{array}{cc} \alpha_{n}\left(t\right) & =\mathrm{atan}2\left(Y_{rn}\left(t\right)-Y_{n}\left(t\right),X_{rn}\left(t\right)-X_{n}\left(t\right)\right)-\Theta_{n}\left(t\right). \end{array}$$

Contrary to previous studies of aperiodic tracking controllers such as [18,27] that use Cartesian coordinates, we work with polar coordinates due to the fact that they allow for reach the target point asymptotically using a time-invariant and smooth feedback controller [36,37]. On the contrary, if the robot is localized with Cartesian coordinates, the control presents some limitations indicated by Brockett’s results [38].

Considering the kinematic model of each robot in the formation, Equation (1), the time derivative of its orientation and distance errors in polar coordinates [37] are:

(4) $$\begin{array}{cc} {\dot{d}}_{n}\left(t\right) & =-v_{n}\left(t\right)\;\cos\left(\alpha_{n}\left(t\right)\right)+v_{rn}\left(t\right)\cos\left(\alpha_{n}\left(t\right)-e_{\Theta_{n}}\left(t\right)\right),\\ {\dot{\alpha }}_{n}\left(t\right) & =-\omega_{n}\left(t\right)+v_{n}\left(t\right)\frac{\sin(\alpha_{n}\left(t\right))}{d_{n}\left(t\right)}-v_{rn}\left(t\right)\frac{\sin(\alpha_{n}\left(t\right)-e_{\Theta_{n}}\left(t\right))}{d_{n}\left(t\right)},\\ {\dot{e}}_{\Theta_{n}}\left(t\right) & =\omega_{rn}\left(t\right)-\omega_{n}\left(t\right). \end{array}$$

The state vector of each unit $\xi_{n}\left(t\right)$ includes the variables presented in the last equation $\xi_{n}\left(t\right)=\left[d_{n}\left(t\right),\alpha_{n}\left(t\right),e_{\Theta_{n}}\left(t\right)\right]$.

**Assumption** **1.** *The virtual leader reference velocities, $v_{rL}\left(t\right)$ and $\omega_{rL}\left(t\right)$, and therefore the reference velocities of each robots, $v_{rn}\left(t\right)$ and $\omega_{rn}\left(t\right)$, are defined piecewise constant and are known beforehand in order to introduce their variations in the self-triggered scheduler.*

### 2.2. Lyapunov Formation Controller

In this section, we present the nonlinear trajectory controller used by each robot in the formation. Inspired by [37], we obtain the following Lyapunov based linear and angular control laws applied to Equation (4):

(5) $$\begin{array}{cc} v_{n}\left(t\right) & =K_{v}d_{n}\left(t\right)\cos\left(\alpha_{n}\left(t\right)\right)+v_{rn}\left(t\right)\cos\left(e_{\Theta_{n}}\left(t\right)\right), \end{array}$$

(6) $$\begin{array}{cc} \omega_{n}\left(t\right) & ={\dot{\Theta }}_{md_{n}}\left(t\right)+v_{md_{n}}\left(t\right)\left(K_{\omega }\left(K_{v}d_{n}\left(t\right)\sin\left(\alpha_{n}\left(t\right)\right)+v_{rn}\left(t\right)\sin\left(e_{\Theta_{n}}\left(t\right)\right)\right)+d_{n}\left(t\right)\sin\left(\alpha_{n}\left(t\right)\right)\right), \end{array}$$

where $\Theta_{md_{n}}$ is the modified desired heading angle of each robot:

(7) $$\Theta_{md_{n}}\left(t\right):=\mathrm{atan}2\left(K_{v}d_{n}\left(t\right)\sin\left(\alpha_{n}\left(t\right)-e_{\Theta_{n}}\left(t\right)\right),v_{rn}\left(t\right)+K_{v}d_{n}\left(t\right)\cos\left(\alpha_{n}\left(t\right)-e_{\Theta_{n}}\left(t\right)\right)\right)+\Theta_{rn}\left(t\right),$$

and $v_{md}$ is equal to:

(8) $$v_{md_{n}}\left(t\right):=\sqrt{v_{rn}{\left(t\right)}^{2}+{\left(K_{v}d_{n}\left(t\right)\right)}^{2}+2v_{rn}\left(t\right)K_{v}d_{n}\left(t\right)\cos\left(\alpha_{n}\left(t\right)-e_{\Theta_{n}}\left(t\right)\right)},$$

and $K_{v}>0$ and $K_{\omega }>0$ are control gains related to the linear and angular velocities, respectively. The closed-loop Lyapunov function for each robot is:

(9) $$V_{n}\left(\xi_{n}\left(t\right)\right)=\frac{1}{2}d_{n}{\left(t\right)}^{2}+1-\cos\left(\Theta_{md_{n}}\left(t\right)-\Theta_{n}\left(t\right)\right).$$

As it is demonstrated in [37], Equation (9) fulfils Lyapunov function properties.

Finally, the Lyapunov function that describes the stability of our road-following formation control is:

(10) $$V\left(\xi \left(t\right)\right)=\sum_{i=1}^{n}V_{n}\left(\xi_{n}\left(t\right)\right).$$

This function also fulfils the Lyapunov conditions because, as demonstrated in [39], a sum of Lyapunov functions is itself a Lyapunov function.

**Remark** **1.** *The choice of Equation (10) is based on the aggregation of the different Lyapunov functions of each unit. This way, the overall stability of the formation is analysed. This is possible because, in the road following strategy, the objectives of each unit are independent of the state of the rest of the units.*

## 3. Lyapunov Based Self-Triggering Control Proposal

First, we introduce briefly the problem formulation of nonlinear aperiodic control. Consider an autonomous nonlinear system:

(11) $$\dot{\xi }\left(t\right)=f\left(\xi \left(t\right),u\left(t\right)\right),$$

where $\xi \left(t\right)\in D_{\xi }\subset R^{n_{x}}$ and $u\left(t\right)\in D_{u}\subset R^{n_{u}}$, both domains containing the origin.

**Assumption** **2.** *There exists a differentiable state feedback law $K:D_{\xi }\to D_{u}$ such that the origin of the closed-loop continuous system*

(12) $$\dot{\xi }\left(t\right)=f\left(\xi \left(t\right),K\left(\xi \left(t\right)\right)\right)$$

*is the unique locally asymptotically stable equilibrium point in $D_{\xi }$.*

If Assumption 2 is fulfilled, we can obtain a Lyapunov function V(ξ(t)) and apply it to the system described in Equation (12) with the following properties [40,41]:

(13) $$\begin{array}{c} \underline{\gamma }\left(||\xi \left(t\right)||\right)\leq V\left(\xi \left(t\right)\right)\leq \bar{\gamma }\left(||\xi \left(t\right)||\right),\\ \dot{V}\left(\xi \left(t\right)\right)=\frac{\partial V(\xi (t))}{\partial \xi }f\left(\xi \left(t\right),K\left(\xi \left(t\right)\right)\right)\leq -\gamma_{1}\left(||\xi \left(t\right)||\right),\\ ∥\frac{\partial V(\xi (t))}{\partial \xi }∥\leq \gamma_{2}\left(||\xi \left(t\right)||\right), \end{array}$$

where $\underline{\gamma },\bar{\gamma },\gamma_{1},\gamma_{2}$ are K-class functions.

**Assumption** **3.** *Assume that:*

*1.*  *The function $f\in C^{l}\left(D_{\xi }\times D_{u}\right)$, with l≥3.*
*2.*  *The functions $\underline{\gamma },\gamma_{1}\in K$ in Equation (13) are such that ${\underline{\gamma }}^{-1},\gamma_{1}$ are Lipschitz continuous on the working compact set $\left(D_{\xi }\right)$. The Lipschitz constants on $D_{\xi }$ of functions ${\underline{\gamma }}^{-1}$ and $\gamma_{1}$ are represented by $L_{{\underline{\gamma }}^{-1}}$ and $L_{\gamma_{1}},$ respectively.*

Between updates, the control signal is held constant with a zero-order hold (ZOH) implementation. When the update times $t_{k}$ are reached, the signal is recalculated with the new measurement:

(14) $$u\left(t\right)=K\left(\xi \left(t_{k}\right)\right),\;t\in [t_{k},t_{k+1}[,\;k\in N.$$

The sampled-data system dynamics with this implementation are:

(15) $$\dot{\xi }\left(t\right)=f\left(\xi \left(t\right),K\left(\xi \left(t_{k}\right)\right)\right),\;t\in [t_{k},t_{k+1}[,\;k\in N.$$

After the introduction of the fundamentals of nonlinear aperiodic control, we present our Lyapunov based self-triggering condition proposal. We design a self-triggering condition assuming that the full state information is available at the measurement instants. Unlike most previous aperiodic control published works [18,21,27,29], we propose a self-triggering condition that triggers the controller taking into account the variation of the Lyapunov function and not the measurement error.

**Definition** **1.** *Semiglobal practical stability [42]: a system $\dot{\xi }\left(t\right)=f\left(\xi \left(t\right),K\left(\xi \left(t\right)\right)\right)$ is said to be semiglobally practically stable if for any (arbitrarily large) compact set $D_{\xi }$ and any arbitrarily small compact set $D_{V_{0}}$ including the origin, every trajectory of the system with $\xi \in D_{\xi }$ is defined for all t∈[0,∞[ and there exists T∈[0,∞[ such that $\xi \in D_{V_{0}}$ for all t∈[T,∞[, where $D_{V_{0}}\subset D_{\xi }$.*

**Theorem** **1.** *Consider that Assumptions 2 and 3 hold for $D_{\xi }$ and $\xi \left(t_{0}\right)\in D_{\xi }$. If the control signal is updated according to the following triggering condition,*

(16) $$t_{k+1}=\min\left\{t>t_{k}|\left(\dot{V}\left(\xi \left(t\right)\right)\geq 0\wedge V\left(\xi \left(t\right)\right)\geq V_{0}\right)\right\},$$

*the system (15) converges asymptotically to the bounded set $D_{V_{0}}$, where $D_{V_{0}}=\left\{\xi \left(t\right)|V\left(\xi \left(t\right)\right)<V_{0}\right\}$.*

**Proof.**  By Theorem 3.2 of [43], the triggering condition (16) presented in Theorem 1 enforces semiglobal practical stability of the system described in Equation (15), as long no Zeno executions are presented. To show that the proposed triggering condition does not introduce Zeno executions, we employ Theorem 4.1 of [29] to guarantee a strictly positive minimum dwell-time between updates.

Theorem 4.1 of [29] is based on the error function g(t):

(17) $$\begin{array}{c} g\left(t\right):=f\left(\xi \left(t\right),K\left(\xi \left(t_{k}\right)\right)\right)-f\left(\xi \left(t\right),K\left(\xi \left(t\right)\right)\right),\;\\ t\;\in \;[t_{k},t_{k+1}[,\;k\in N. \end{array}$$

Using this error function, the dynamics of the sample-data system (15) are rewritten as:

(18) $$\dot{\xi }\left(t\right)=f\left(\xi \left(t\right),K\left(\xi \left(t\right)\right)\right)+g\left(t\right),\;t\in [t_{k},t_{k+1}[,\;k\in N,$$

and the following sampling rule is proposed by [29]:

(19) $$t_{k+1}=min\{t>t_{k}\;|\;||g\left(t\right))||>e_{V_{0}}\}.$$

This way, Equation (19) ensures semiglobal practical stability of the closed-loop system for any $e_{V_{0}}>0$ and guarantees a positive minimum dwell-time if Assumptions 2 and 3 hold for any (arbitrarily large) compact set $D_{\xi }$ and $\xi \left(t_{0}\right)\in D_{\xi }$.

If we can proof that condition described in Equation (16) generates equal or greater inter-execution times than (19) for some selection of $e_{V_{0}}>0$, then, by virtue of Theorem 4.1 of [29], we obtain the desired result: the existence of a strictly positive minimum dwell-time, and thus no Zeno execution is possible. Observe that:

(20) $$\begin{array}{c} \dot{V}\left(\xi \left(t\right)\right)=\frac{\partial V(\xi (t))}{\partial \xi }f\left(\xi \left(t\right),K\left(\xi \left(t_{k}\right)\right)\right)=\\ \frac{\partial V(\xi (t))}{\partial \xi }f\left(\xi \left(t\right),K\left(\xi \left(t\right)\right)\right)+\\ \frac{\partial V(\xi (t))}{\partial \xi }\left(f\left(\xi \left(t\right),K\left(\xi \left(t_{k}\right)\right)\right)-f\left(\xi \left(t\right),K\left(\xi \left(t\right)\right)\right)\right)\leq \\ -\gamma_{1}\left(||\xi \left(t\right)||\right)+\gamma_{2}(||\xi \left(t\right)||)||g\left(t\right)||, \end{array}$$

which, together with Equation (19), implies:

(21) $$\begin{array}{c} \dot{V}\left(\xi \left(t\right)\right)\leq -\gamma_{1}\left(||\xi \left(t\right)||\right)+\gamma_{2}{\left(||\xi \left(t\right)|\right|}_{L_{\infty ,k}})e_{V_{0}}. \end{array}$$

Thus, Equation (19) enforces the following implication:

(22) $$\dot{V}\left(\xi \left(t\right)\right)<0\;\forall t,\;\mathrm{if}\;||\xi \left(t\right)||>\gamma_{1}^{-1}\left(\gamma_{2}{\left(||\xi \left(t\right)|\right|}_{L_{\infty ,k}})e_{V_{0}}\right).$$

Inspecting the triggering condition (16), we observed that it *only* demands new updates if:

(23) $$\dot{V}\left(\xi \left(t\right)\right)\geq 0\;\wedge \;V\left(\xi \left(t\right)\right)>V_{0}.$$

Thus, from Equation (13), we have that Equation (16) forces updates when:

(24) $$\dot{V}\left(\xi \left(t\right)\right)\geq 0\;\wedge \;||\xi \left(t\right))||\geq {\bar{\gamma }}^{-1}\left(V_{0}\right).$$

Select now an $e_{V_{0}}$ such that:

(25) $$\gamma_{1}^{-1}(\gamma_{2}{\left(||\xi \left(t\right)|\right|}_{L_{\infty },k})e_{V_{0}})\leq {\bar{\gamma }}^{-1}\left(V_{0}\right).$$

Such an $e_{V_{0}}>0$ always exists as long as ${\left||\xi \left(t\right)|\right|}_{L_{\infty },k}$ is upper bounded, from the properties of $K_{\infty }$ functions and $V_{0}>0$. Note that ${\left||\xi \left(t\right)|\right|}_{L_{\infty },k}$ is upper bounded as V(ξ(0))<∞ and thus due to Equation (16) V(ξ(t))<∞ for all positive times, which by Equation (13) implies the required boundedness.

Finally, by virtue of Equation (25), we have that, when triggering function (24) takes place, the triggering condition from Equation (19) is certainly violated. ☐

**Figure 2:**
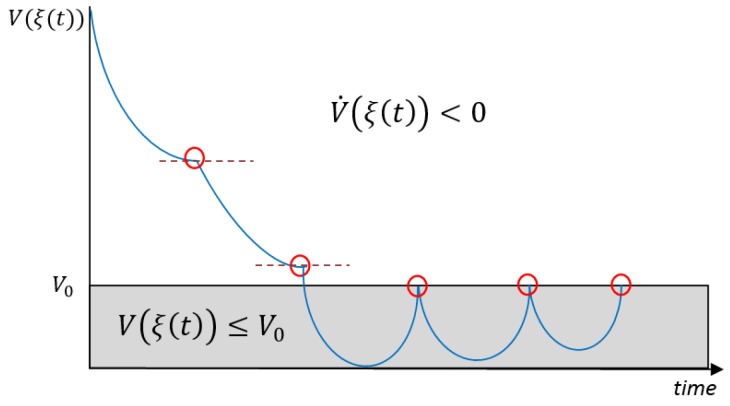
Description of the Theorem 1 triggering condition (16). If the Lyapunov function is greater than $V_{0}$, the system is updated every time the derivative of the Lyapunov function is non-negative. If the system converges to the invariant set defined by $V_{0}$, the system is triggered only when the Lyapunov function reaches the threshold $V_{0}$.

Figure 2 describes graphically the Theorem 1 triggering condition.

**Remark** **2.** *Theorem 1 guarantees $\dot{V}\left(\xi \left(t\right)\right)<0$ when the system is outside the invariant set $D_{V_{0}}$. The choice of $V_{0}$ establishes a trade-off between the invariant set $D_{V_{0}}$ and the inter-execution times $[t_{k},t_{k+1}[$. Decreasing the value of $V_{0}$ reduces the size of the set $D_{V_{0}}$ as well as the inter-execution times.*

**Remark** **3.** *To find a dwell-time, we use Theorem 4.1 from [29] because our triggering condition (16) presents no inter-execution times lower than [29] outside of the bounded set defined by $D_{V_{0}}$.*

Finding a lower bound of the inter-sampling time $t_{min}$ is still an open issue [29]. Nevertheless, using Remark 3, we guarantee that $t_{min}$ is positive and, in order to avoid Zeno-executions, we force a $t_{min}$, taking into account the hardware constraints.

We design two different triggering strategies, one synchronous and centralized and the other asynchronous and decentralized. For the centralized strategy, we evaluate the triggering condition (16) with the global Lyapunov function of the formation (10). In this case, the triggering instants are:

(26) $$t_{k+1}=\min\left\{t>t_{k}|\left(\dot{V}\left(\xi \left(t\right)\right)\geq 0\wedge V\left(\xi \left(t\right)\right)\geq V_{0}\right)\right\}.$$

We also implement a decentralized solution for the formation, in which each robot unit triggers its own events asynchronously. Formation stability is also guaranteed because this strategy is more trigger demanding than the global one:

(27) $$t_{nk+1}=\min\left\{t>t_{n_{k}}|\left(\dot{V_{n}}\left(\xi_{n}\left(t\right)\right)\geq 0\wedge V_{n}\left(\xi_{n}\left(t\right)\right)\geq V_{0}/n\right)\right\}.$$

**Remark** **4.** *For the decentralized triggering strategy (27), the threshold $V_{0}$ is divided by the number of robot units, as can be seen in the equation. Thus, we guarantee that the formation converges asymptotically to the same bounded set as when we implement the centralized condition (26).*

Another important parameter of STC is $t_{max}$, which means the maximum time the system is allowed to run in open loop and it is a design parameter [26]. This implies that, if the inter-execution time $\left(t_{k+1}-t_{k}\right)$ obtained with the triggering condition is greater than the $t_{max},$ the next update instant $t_{k+1}$ is modified to $t_{k}+t_{max}$. This parameter is used to reduce the time that the system works in open loop, which is the main disadvantage of STC with respect to ETC. This way the designer can fix this parameter to limit the possible uncertainties or disturbances that can affect the system.

## 4. Simulation Results

**Figure 3:**
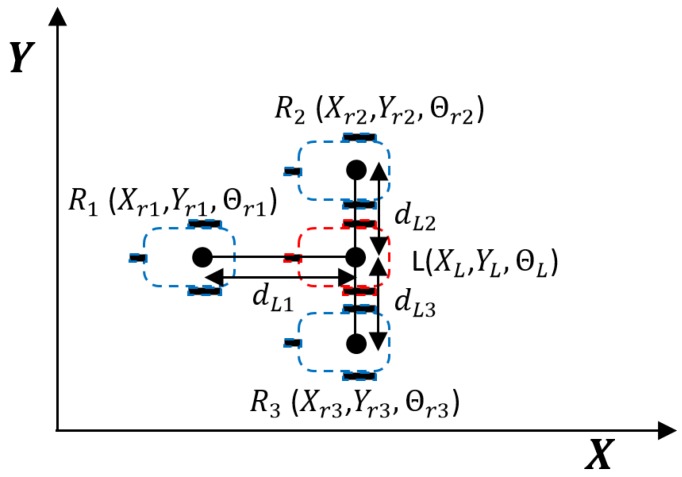
Main variables describing the formation strategy: in blue color the reference poses of each robot $R_{n}$ $\left(X_{rn},Y_{rn},\Theta_{rn}\right)$, which are generated according the position of the Virtual Leader *L* $\left(X_{L},Y_{L},\Theta_{L}\right)$ in red.

In this section, we describe a simulation of formation control of three robots based on the previously mentioned robot kinematic model (1). A virtual leader tracks the desired nonlinear trajectory without error and determines the position of each robot in the formation and its velocity references using a road-following formation strategy, as described in Figure 3.

The distance between the reference robots and the virtual leader is determined by $d_{Ln}$. In our example, we set $d_{L1}=1\;m$ and $d_{L2}=d_{L3}=0.4\;m$. To implement the spatially coordinated road-following formation [35], delayed velocity references of the Virtual Leader are sent to the robots depending on the longitudinal distance from the robot to the Virtual Leader, and adjusted linear velocity references for the Virtual Leader are sent to the robots depending on the curvature of the route in each track and the lateral distance from the robot to the Virtual Leader. In the formation shown in Figure 3, Robot 1 receives the delayed velocity reference and Robot 2 and Robot 3 the references adjusted for the curvature of each track. Note that, with our formation strategy, the relative positions of the vehicles at a given point in time are not coordinated at every moment, but the trajectories of the vehicles are always spatially aligned.

**Figure 4:**
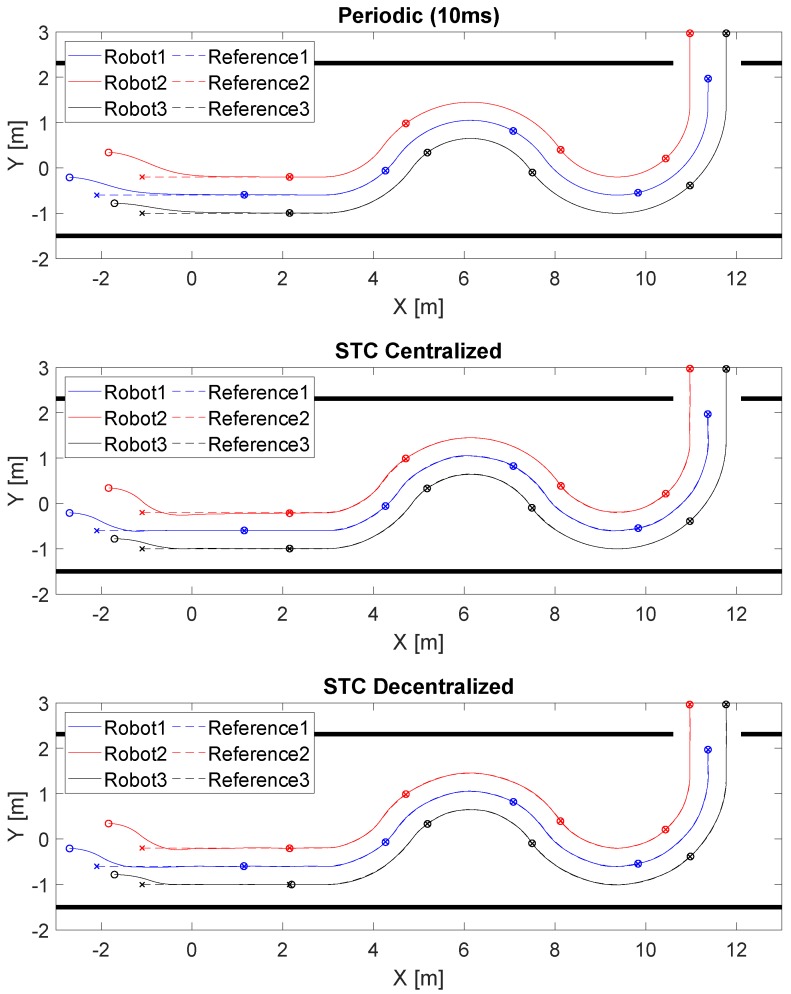
Nonlinear trajectory tracked by the formation of three robotic units. Robot 1 is represented in blue, Robot 2 in red and Robot 3 in black. The route followed by each robot is presented with a continuous line, the reference with a discontinuous line, and every 13 s the punctual position of each of them is shown with a circle and an x, respectively. The top figure represents the periodic implementation, the middle one the STC with centralized triggering (26) and the bottom the STC with decentralized triggering (27).

The control parameters of the robots are: $K_{v}=0.8$, $K_{w}=0.05$, $t_{min}=10\;\mathrm{ms}$, $t_{max}=5\;s$ and $V_{0}=10^{-4}$. The initial robot locations ([m,m,rad]) are $F_{1}=\left[-2.70;-0.21;0\right]$, $F_{2}=\left[-1.84;0.34;0\right]$ and $F_{3}=\left[-1.71;-0.78;0\right]$. The initial formation reference points are $R_{1}=\left[-2.1;-0.6;0\right]$, $R_{2}=\left[-1.1;-0.2;0\right]$ and $R_{3}=\left[-1.1;-1;0\right]$. The nonlinear trajectory tracking by the formation of robots is shown in Figure 4. We obtained good tracking performance with all the control solutions tested: periodic (Ts=10 ms), STC with centralized triggering (26) and STC with decentralized triggering (27). The sampling period is chosen according to the hardware restrictions imposed by the P3DX robots and the minimum time between events of the STC control (*Ts* = 10 ms).

**Figure 5:**
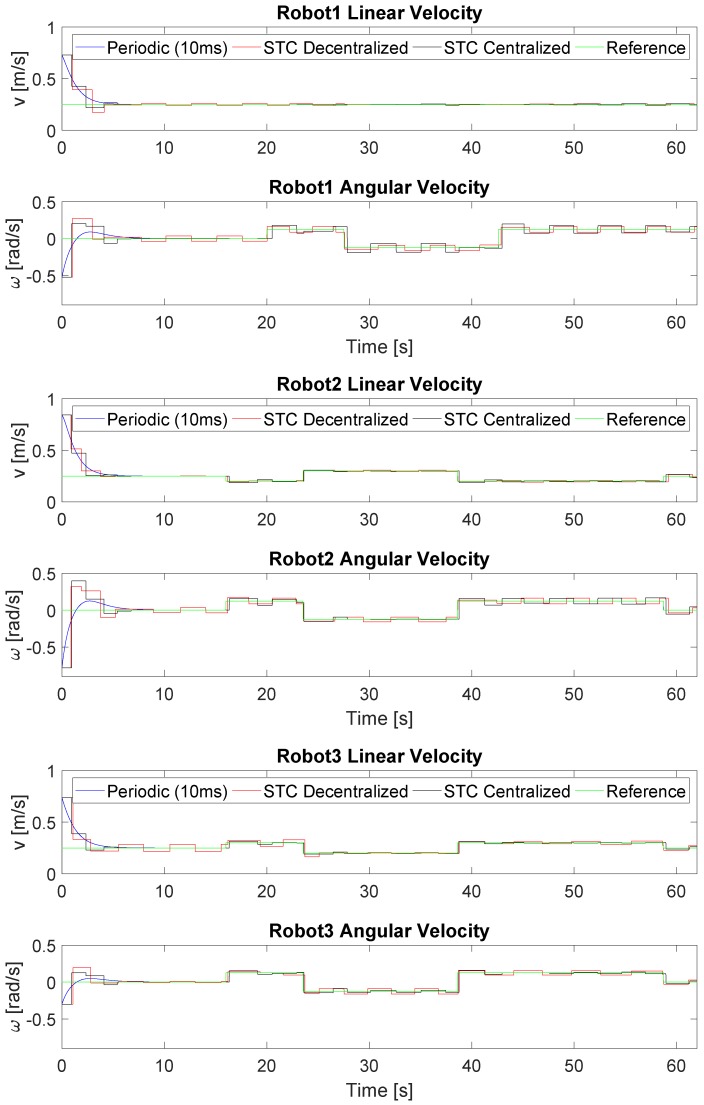
Linear and angular velocity references and commands of the formation. Robot 1 is represented in top figure, Robot 2 in middle one and Robot 3 in the bottom. The velocity done by each robot is presented in blue for the periodic implementation, in red for the STC with decentralized triggering (27) and in black for the centralized triggering (26). The velocity reference of each unit is presented in green.

Figure 5 shows the linear and angular velocity references and commands of the formation applying the three different strategies. In the case of periodic control, there is a practically continuous evolution in which the error is corrected gradually until being accurately linked to the reference at the cost of a large number of updates compared to the STC strategies. In the case of the STCs, changes in the velocity commands only occur at specific moments in time. For the centralized solution, each robot update is activated at the same time, with the corresponding additional reduction in the number of updates, while, for the decentralized strategy, this is performed at different moments according to triggering conditions (26) and (27), respectively.

**Figure 6:**
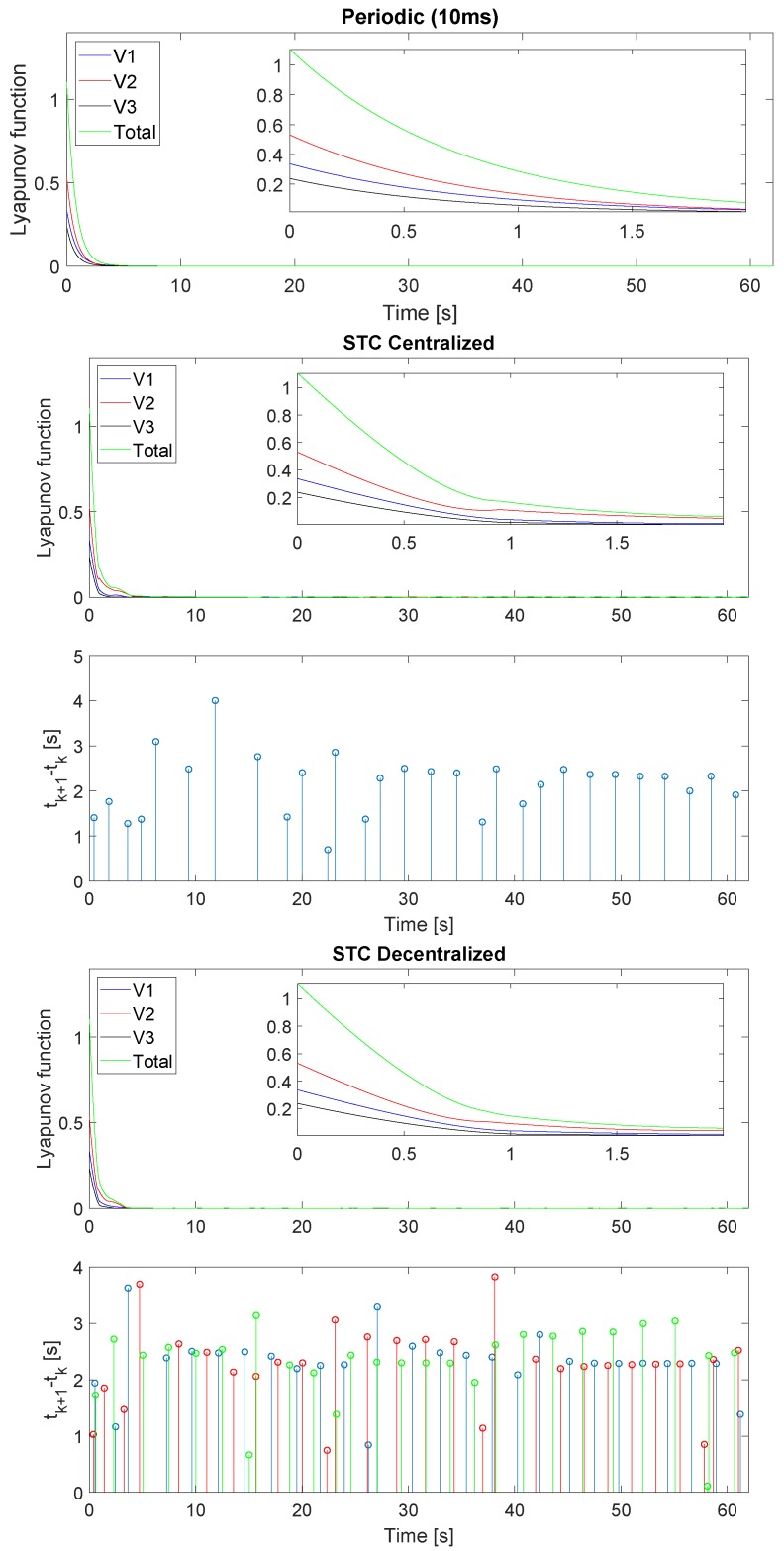
Lyapunov function of the formation including a zoom of the first 2 s and inter-execution times for STC startegies. Robot 1 is drawn in blue, Robot 2 in red, Robot 3 in black and the Formation in green. Top figure represents the periodic implementation, the middle one the STC with centralized triggering (26) and the bottom the STC with decentralized triggering (27).

Figure 6 describes the evolution of the Lyapunov function of the formation (10), the Lyapunov function of each robot (9) and the inter-execution-times of the STC implementations. As can be seen, the system reaches the equilibrium point properly with all three strategies. In the case of decentralized triggering (27), the gradient of the Lyapunov function of all the robots is negative at all times in the transient regime. In contrast, in the case of centralized triggering (26), there are specific moments in which a robot Lyapunov function slope may be positive; however, the formation slope is negative at all times.

Table 1 quantifies the number of updates and Root Mean Squared (RMS) value for distance error (2) of the formation with the control strategies tested. With the designed control, STC significantly reduces the number of updates in the control signals applied to the robot, as well as achieving a performance of the same magnitude as periodic implementation. With centralized triggering, there is a greater reduction in the number of updates because the commands are sent to the three robots at the same time. On the other hand, with decentralized triggering, the event is evaluated at the precise moment for each robot, but at the cost of a larger number of updates.

We also present a statistical study to validate the controllers. In this study, we carry out 100 simulations with the different strategies. We use a random combination for the initial pose conditions, all of them within a radius of 2 m from the previous starting positions, $F_{1}$, $F_{2}$ and $F_{3}$. Table 2 shows the mean and standard deviation for performance and updates of each control technique. For the case under study, our STC solutions provides the best results with a relevant reduction in the controller updates comparing with the periodic implementation, without diminishing performance.

## 5. Remote Centre Task Scheduler

**Figure 7:**
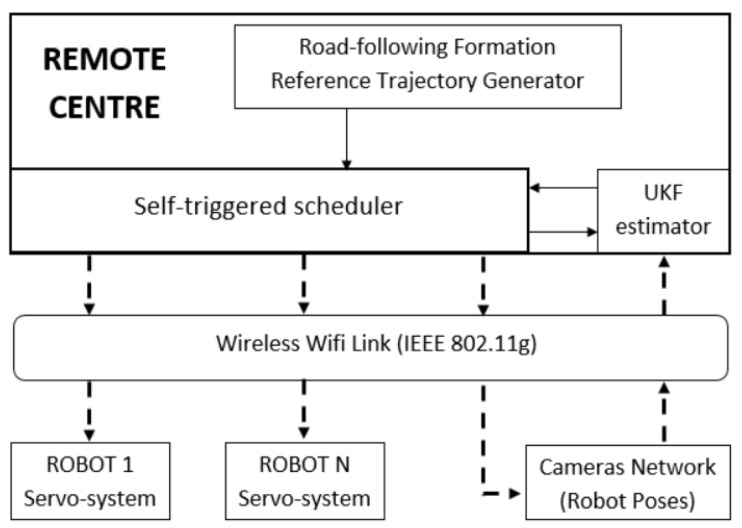
Description of the main elements in our implementation scenario: the remote centre, the robots formation, the camera network and the wireless communication channel. The remote centre carry out the principal tasks: reference trajectory generation of each robot according to the road-following strategy, unscented Kalman filter(UKF) and self-triggered control based on Lyapunov functions for asynchronous request of measurements and actuations on the robots.

In this section, we describe one of the most critical aspects of our remote centre: the design of our task scheduler and its interaction with the robots and the wireless sensor nodes. To enable a better understanding of the experimental validation of the aperiodic strategies applied to trajectory tracking of a robot formation, we detail the role of the remote center. Figure 7 shows the main elements of our implementation scenario.

The implementation scenario has four main elements:

1. A non-holonomic mobile robot formation. Each robot locally implements a periodic servosystem for linear and angular velocity tracking.
2. A set of sensor nodes covers the entire experimental area and provides each robot with pose information using computer vision.
3. An IEEE 802.11g standard wireless network that links the remote centre to the robots and the set of sensor nodes.
4. A remote centre that performs the principal tasks: trajectory generation for the virtual leader considered the reference for the road-following formation, trajectory generation for each real robot with respect to the virtual leader, new measurement request to the camera network, pose estimation of each robot unit based on the UKF, and application of the self-triggered control strategy.

### 5.1. Delay Compensation

In the network control system practical implementation, the time delays must be taken into account. Leveraging one of the main strengths of STC, namely that the next sampling instant is known in advance, we designed a channel delay compensation strategy inspired by [30].

We consider the following delays:

1. $\tau_{t}$ is the maximum network delay, it is the maximum time to transmit a message via the wireless communication network.
2. $\tau_{s}$ is the maximum sensor delay, i.e., the time between the start of a measurement acquisition in the sensor node and the instant it is ready to be sent to the remote centre. This time includes image acquisition and processing.
3. $\tau_{c}$ is the control computing time of the remote centre.
4. $\tau_{r}$ is the dominant constant time that characterizes the robot dynamics.

**Figure 8:**
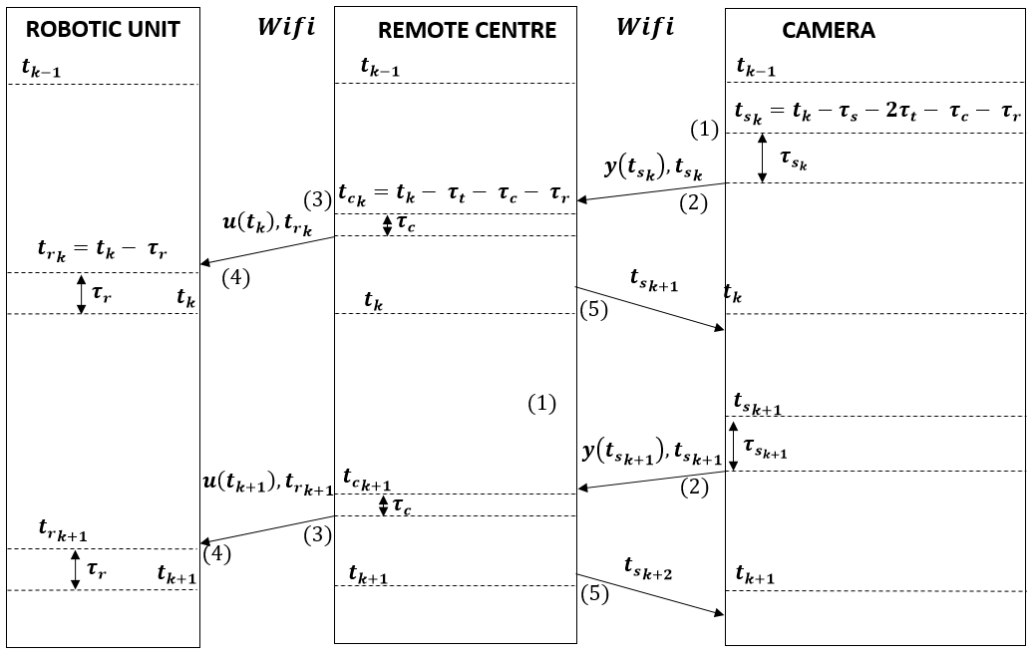
Communication protocol between the remote centre, sensor and robot, with the delay compensation strategy.

Quantifing these delays requires clock synchronization between the sensors, robots and remote centre. We decided to use Network Time Protocol (NTP) due to it allowing a correct synchronization in the order of magnitude of our time step (Δ=10ms). In Figure 8, we describe our strategy to compensate delays and robot dynamics:

The main idea of the global delay compensation strategy is to pre-calculate robot control actions using the predictive capability of the self-triggered control. This strategy involves the following five steps:

1. At time instant $t_{s_{k}}$, the cameras start the measurement process with image acquisition. This time is previously indicated to the cameras by the STC of the remote centre. Computation of this time is explained in Step 5.
2. When the pose measurement ($t_{s_{k}}$) is ready, the cameras send it and the acquisition time ($t_{s_{k}}$) to the remote centre.
3. At time instant $t_{c_{k}}$, the UKF of the remote centre corrects the prediction of the states for time instant $t_{sk}$ with the measurement sent by the camera ($y(t_{s_{k}})$). Next, the UKF predicts the states at time instant $t_{k}$ and sends this information to the STC controller. With this information, the STC generates linear and angular speed commands for each robot ($u(t_{k})$) and computes the next update instant ($t_{k+1}$). The control signal ($u(t_{k})$) and the application time are sent to the robot ($t_{r_{k}}$)
4. At time instant $t_{r_{k}},$ the control signal is applied to the robot; thus, the desired control signal is reached at time instant $t_{k}$ compensating the robot dynamics.
5. After sending the control signal to the robots, the remote centre sends the next measurement acquisition time to the cameras ($t_{s_{k+1}}$). This time is computed taking into account the next sampling instant $t_{k+1}$ and all the delays ($t_{s_{k+1}}=t_{k+1}-\tau_{s}-2\tau_{t}-\tau_{c}-\tau_{r}$).

**Remark** **5.** *In certain situations, the time between update times ($t_{k+1}-t_{k}$) will be less than the sum of all the delays ($\tau_{s}+2\tau_{t}+\tau_{c}+\tau_{r}$). In this case, the system will work with the state prediction provided by the UKF until a new measurement is available to include in the UKF correction.*

### 5.2. Control Design Dependent on State Estimation

We implement an UKF which takes care of bounding the estimation error between measurement. Our self-triggered controller computes the update times based on the output of this filter. The principal drawbacks of this implementation is that bounded peaks may be obtained in the Lyapunov function when the estimation error grows; consequently, we have designed an aperiodic controller that minimizes this effect by guaranteeing practical stability of the system instead of asymptotic stability. Thus, our strategy achieves longer times between control updates than periodic or aperiodic alternatives aimed at guaranteeing asymptotic or exponential stability.

## 6. Experimental Tests

**Figure 9:**
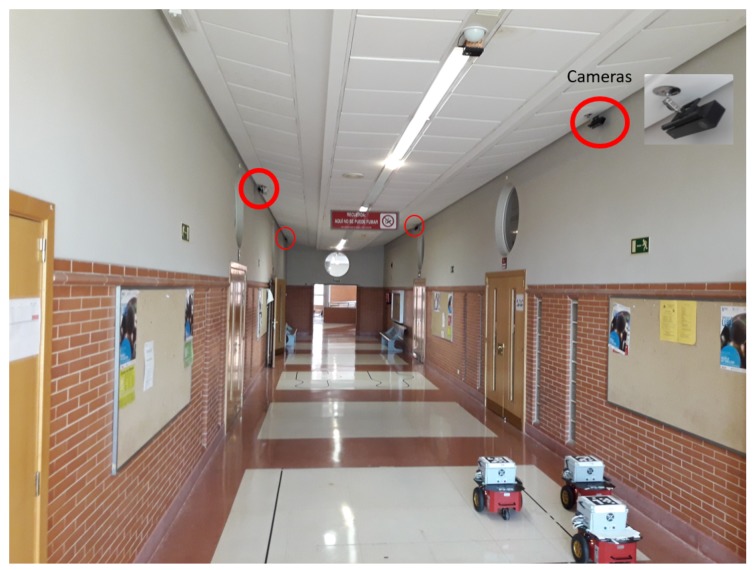
Picture of the working area with four Kinect 2 camera sensor and the formation of three P3DX robot wirelessly controlled by a miniPC.

The tests are carry out with three Pioneer P3DX robots (Product specs can be found at http://www.mobilerobots.com/ResearchRobots/PioneerP3DX.aspx) with additional hardware elements [44]. The remote centre control is implemented in a NUC5i3RYH mini PC (Model number NUC5i3RYH. Product specs can be found at https://www.intel.com/content/www/us/en/nuc/nuc-kit-nuc5i3ryh-brief.html). The test area is sensed by four Kinect RGB cameras (Microsoft, Redmond, WA, USA) connected to an identical mini PC. All mini PCs run Ubuntu 12.04, as an operating system. An AprilTag marker is situated on the top of each P3DX robot to obtain its pose using the AprilTags fiducial system presented in [45]. Figure 9 shows this experimental scenario.

### Results

Below, we present three experimental tests with the road-following formation strategy, the control parameters and the initial conditions described in Section 4. The control parameters of each robots are: $K_{v}=0.8$, $K_{w}=0.05$, $t_{min}=10\;\mathrm{ms}$, $t_{max}=5\;s$ and $V_{0}=10^{-4}$. The initial robot locations are $F_{1}=\left[-2.70;-0.21;0\right]$, $F_{2}=\left[-1.84;0.34;0\right]$ and $F_{3}=\left[-1.71;-0.78;0\right]$. The initial formation points are $R_{1}=\left[-2.1;-0.6;0\right]$, $R_{2}=\left[-1.1;-0.2;0\right]$ and $R_{3}=\left[-1.1;-1;0\right]$.

**Figure 10:**
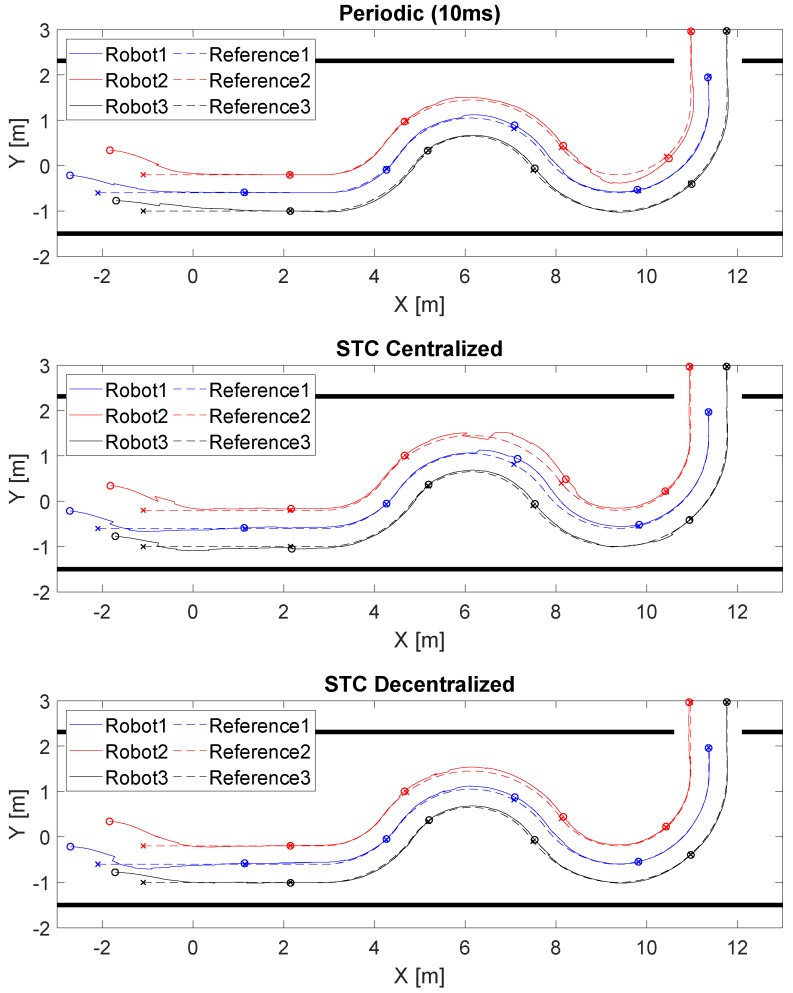
The nonlinear, trajectory tracking by the formation of three robotic units. Robot 1 is represented in blue, Robot 2 in red and Robot 3 in black, the route done by each robot is presented with a continuous line, the reference with a discontinuous line, and every 13 s the punctual position of each of them is shown with a circle and an x, respectively. The top figure represents the periodic implementation, the middle one the STC with centralized triggering (26) and the bottom the STC with decentralized triggering (27).

Figure 10 depicts the formation of P3DX robot trajectory tracking for the three solutions tested (periodic (Ts=10 ms), STC with centralized triggering (26) and STC with decentralized triggering (27). We include a video, see Supplementary Materials, showing the experimental test using the STC with decentralized triggering). If we compare it with Figure 4, it can be seen that performance deteriorates a little because the simulation assumes a perfect communication channel, robot models based only on kinematics and position sensing without error.

**Figure 11:**
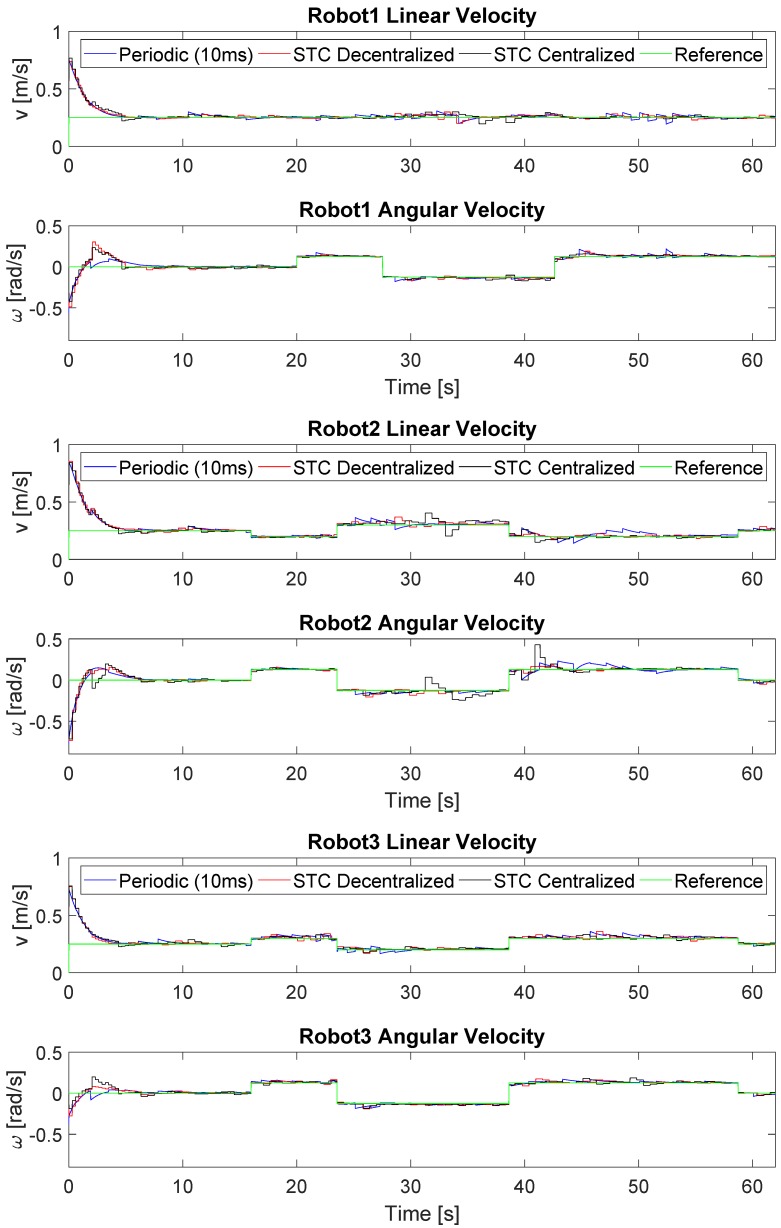
Linear and angular velocity references and commands of the formation. Robot 1 is represented in the top figure, Robot 2 in the middle one and Robot 3 in the bottom. The velocity done by each robot is presented in blue for the periodic implementation, in red for the STC with decentralized triggering (27) and in black for the centralized triggering (26). The velocity reference of each unit is presented in green.

Figure 11 shows the linear and angular velocity reference and commands of the formation for each robot applying the three different strategies. As in simulation, the STC implementations applied few updates in the velocity commands; these updates are activated at the same time in the centralized solution and at different moments in the decentralized solution.

**Figure 12:**
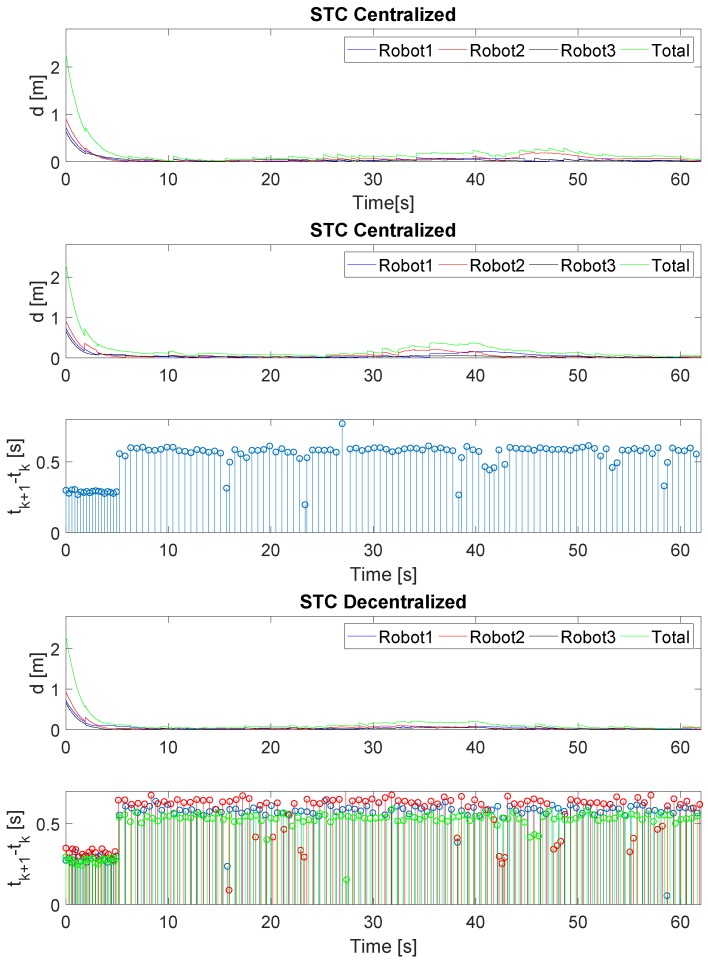
Lyapunov function of the formation and inter-execution times for STC strategies. Robot 1 is represented in blue, Robot 2 in red, Robot 3 in black and the Formation in green. The top figure represents the periodic implementation, the middle one the STC with centralized triggering (26) and the bottom the STC with decentralized triggering (27).

Figure 12 describes the evolution of the formation distance error (2), the error of each robot (9) and the inter-execution times for the STC strategies. As can be seen, the system is bounded around the null distance error with the three strategies. When a new measurement is obtained, the UKF corrects its prediction and a small jump appears in the distance error.

Table 3 quantifies the number of updates and the Root Mean Squared (RMS) value for distance error (2) of the formation with the different control strategies. The experimental test results follow the same trend as those for simulation. Thus, STC significantly reduces the number of changes in the control signals applied to the robot, as well as achieving a performance of the same magnitude as periodic implementation.

## 7. Conclusions

This paper describes the design and implementation of a self-triggered remote road-following formation controller applied to nonholonomic robots tracking nonlinear trajectories using an external positioning sensor network.

We design a novel self-triggered Lyapunov-based controller, using a dual stability approach in order to guarantee practical stability. Unlike most previous work in the field of aperiodic control, the measurement error is not taken into account when the controller is triggered, thus achieving longer inter-execution times as the solution is less conservative. This triggering condition is implemented adopting both a centralized and a decentralized approach, highlighting the advantages of each strategy.

STC solutions have been implemented taking into account practical drawbacks such as communication network delays, actuation time delays due to robot dynamics, and acquisition and processing times associated with the camera sensor to obtain the pose of each unit. To minimize the effect of these problems on the stability and performance of the closed loop system, we designed and implemented a delay compensation strategy. This strategy leverages the predictive capability of self-triggered control to pre-calculate control actions and compensate delays.

To validate the theoretical proposal, several experimental tests are conducted using the designed self-triggered controllers and a periodic implementation. We report simulation results, showing that our control solution significantly reduces the need for communication in comparison with periodic implementations while preserving the desired tracking performance. To validate the proposal, we also perform experimental tests with a formation of three P3DX robots remotely controlled through an IEEE 802.11g wireless network with a mini PC, in which robot pose is detected by a set of camera sensors connected to the same wireless network. The results obtained for our self-triggered control solution indicate that using our aperiodic proposals rather than periodic solution yields a significant reduction in the number of updates without degrading performance, rendering this kind of control solution especially interesting for networked control systems and wireless sensor network integration.

For future work, we are going to consider the design of an ETC to implement strategies of obstacle avoiding combined with collision avoiding between the different units of the formation.

## Figures and Tables

**Table 1:** Comparison of the update number and RMS value of distance error concerning the different control strategies for nonlinear, trajectory tracking (see Figure 4): periodic implementation, STC with centralized triggering (26) and STC with decentralized triggering (27).

	Periodic [10 ms]				STC Centralized (26)				STC Decentralized (27)			
	R1	R2	R3	**Formation**	R1	R2	R3	**Formation**	R1	R2	R3	**Formation**
Updates	6500	6500	6500	**6500**	29	29	29	**29**	29	30	28	**87**
$d_{RMS}\;\left[m\right]$	1.31	1.39	1.04	**3.74**	1.12	1.58	0.92	**3.62**	1.19	1.39	1.37	**3.95**

**Table 2:** Comparison of the average (AVG) and the standard deviation (STD) values of update number and RMS value of distance after 100 simulation results concerning the different control strategies for nonlinear, trajectory tracking: periodic implementation, STC with centralized triggering (26) and STC with decentralized triggering (27).

	Periodic [10 ms]				STC Centralized (26)				STC Decentralized (27)			
	R1	R2	R3	**Formation**	R1	R2	R3	**Formation**	R1	R2	R3	**Formation**
AVG Updates	6200	6200	6200	**6200**	32.70	32.70	32.70	**32.70**	30.37	30.89	30.22	**91.48**
STD Updates	0	0	0	**0**	4.78	4.78	4.78	**14.34**	3.26	3.69	6.99	**10.51**
AVG $d_{RMS}\;\left[m\right]$	3.54	3.56	3.82	**10.91**	3.35	3.23	3.16	**9.74**	3.17	3.26	3.28	**9.71**
STD $d_{RMS}\;\left[m\right]$	1.68	1.71	1.84	**4.96**	1.25	1.23	1.27	**3.64**	1.25	1.23	1.28	**3.69**

**Table 3:** Comparison of the update number and RMS value of distance concerning the different control strategies for trajectory tracking, including the periodic implementation, the STC with centralized triggering (26) and the STC with decentralized triggering (27).

	Periodic [10 ms]				STC Centralized (26)				STC Decentralized (27)			
	R1	R2	R3	**Formation**	R1	R2	R3	**Formation**	R1	R2	R3	**Formation**
Updates	6500	6500	6500	**6500**	125	125	125	**125**	123	119	136	**378**
$d_{RMS}\;\left[m\right]$	2.89	4.61	3.12	**10.62**	2.94	4.90	3.72	**11.56**	2.24	3.34	3.05	**8.63**

## Supplementary Materials

A video showing the results of one of the performed experimental tests is available online at http://www.geintra-uah.org/idi/demostraciones/demostraciones#self19.
